# Re-visiting genetic background of some native Turkish sheep populations: bottleneck and migration

**DOI:** 10.1007/s11250-025-04520-6

**Published:** 2025-06-17

**Authors:** Bahar Argun Karsli, Eymen Demir, Umit Bilginer, Murat Soner Balcioğlu, Taki Karsli

**Affiliations:** 1 Department of Agricultural Biotechnology, Faculty of Agriculture, Eskişehir Osmangazi University, Eskişehir, 26160 Türkiye; 2 Department of Animal Science, Faculty of Agriculture, Akdeniz University, Antalya, 07070 Türkiye; 3 Department of Animal Science, Michigan State University, East Lansing, MI 48824 USA; 4 Department of Animal Science, Faculty of Agriculture, Eskişehir Osmangazi University, Eskişehir, 26160 Türkiye

**Keywords:** Anatolian sheep, Bottleneck, Conservation, Demographic history, Migration, SSR

## Abstract

This study aimed to investigate genetic bottleneck effects and migration events among four native Turkish sheep breeds, namely Güney Karaman (GKR), Karakaş (KRK), Kangal (KNG), and Norduz (NRD). After genotyping a total of 120 animals with 28 highly polymorphic microsatellite loci, the genetic bottleneck was assessed by the Wilcoxon test under different mutation models, while population splits and migration events were investigated by the TreeMix algorithm. Wilcoxon sign rank test under the two-phased mutation model (TPM) and the mode-shift indicator based on the distribution of allele frequencies evidenced a lack of genetic bottleneck in four Anatolian sheep breeds. This finding implies that the studied sheep breeds have maintained their effective population size in the recent past. Similarly, the estimated values of effective population size were higher than the number of sampled animals, indicating that they were descendants of ancestral populations with higher sample sizes. The TreeMix algorithm revealed that the NRD was genetically distinct from the other breeds, while there was migration from NRD to KRK and GKR with a rate of 0.0096. The highest migration rate (0.0176) was detected from the KNG to the GKR breed. The results of this study are expected to assist breeders in taking necessary precautions for sustainable production in the future and to facilitate ongoing conservation programs. Indeed, breeders are encouraged to utilize both microsatellites and high-throughput genomic tools such as SNP arrays and next-generation sequencing technologies to foresee the trend in effective population size and genetic bottleneck effects in local sheep populations. Besides, geographic isolation and pure breeding of the NRD breed should be considered in conservation programs to eliminate inbreeding depression and genetic bottleneck in the future.

## Introduction

Being still considered sacred in several cultures, sheep and goats have played a key role in the civilization of humankind since their domestication. They not only offer animal-derived products such as meat and milk, which are essential for a healthy and balanced diet for societies, but also the weaving and textile industry make use of raw materials such as wool and leather obtained from small ruminant rearing (Alberto et al. 2018; Demir et al. 2022). Compared to developed countries, sheep rearing is more beneficial in developing countries such as Türkiye due to the fact that it covers a main part of breeders’ incomes and allocates new opportunities for employment in rural areas (Yildiz and Aygun 2021). Indeed, sheep rearing has been a part of daily life in Türkiye leading to a grassland-based lifestyle called the nomadic breeding system (Karsli et al. 2020). Türkiye ranks amongst the leading sheep producers in the Middle East by holding approximately 45 million heads, of which more than 91% of the population are native sheep (FAO 2022; TSI 2023). Besides, Türkiye, homing to nearly 30 indigenous sheep breeds and ecotypes, makes a significant contribution to the world's animal genetic resources. Numerous breeds are now under the national conservation program because they are on the brink of extinction (GDARP 2009; Karsli et al. 2020). Being distributed across the country but mainly reared in central Anatolia, Akkaraman (AKR) constitutes more than 40% of the total sheep population. AKR is thought to have numerous ecotypes such as GKR, NRD, KRK, KNG, and Şavak which are believed to develop adaptations to different geographical regions. Of these varieties, GKR is known to have experienced a significant reduction in population size over the past 30 years forcing the Ministry of Agriculture and Forestry to initiate a conservation program. Mainly distributed to the southern part of Anatolia, this breed is well-adapted to high temperatures. NRD and KRK, on the other hand, are reared in the eastern part of Anatolia including Van, Hakkari, and Diyarbakır provinces. Being reared in Sivas and nearby provinces, KNG has been obtained from the AKR breed via selection studies to increase body weight (Karsli et al. 2011, 2020; Yagci et al. 2020).

Türkiye is part of the"Fertile Crescent", one of the sheep domestication areas which make the country a historical trade and migration route. This situation has created a great genetic diversity in Türkiye's native livestock breeds and ecotypes (Demir et al. 2023). However, high-yield genotypes obtained as a result of developments in animal breeding in the last century have become dominant in Türkiye as well as all over the world by creating great pressure on native livestock breeds and causing a decrease in genetic diversity. In addition, non-systematic crossbreeding studies carried out by breeders have also caused genetic erosion in native breeds (Demir and Balcioglu 2019).

Genetic diversity, which is an important assurance for sustainable agricultural activities, contributes significantly to long-term sustainability of livestock production (Ghildiyal et al. 2023). Animal genetic resources and their genetic diversity are indispensable elements to ensure the continuity of animal production under environmental conditions that are likely to change in the future (Soysal et al. 2020). For sustainability in domestic livestock breeds and ecotypes, which are a major component of animal genetic resources, it is important to determine the genetic diversity in existing populations and the underlying causes of this diversity (DeWoody et al. 2021). Genetic diversity in populations has been affected by various events such as mutation, adaptation, isolation, migration, and breeder preferences in the period from domestication to the present day. A decrease in genetic diversity leads to increased susceptibility to diseases and decreased ability to adapt to changing environmental conditions. In addition, the founder effect may cause the frequency of various alleles to change in the population due to the decrease in population size due to various events in the past.

Microsatellite markers are valuable tools that are extensively used in analyses of genetic diversity, phylogenetic relationships, population structure, bottlenecks, and migration in livestock species and breeds (Silvestre et al. 2015; Karsli and Balcıoğlu 2019; Demir and Balcıoglu 2019; Karsli et al. 2020). Sudden reduction in population size may cause a decreased genetic diversity, which is defined as variations in the number of repeat motifs in microsatellite genotyping. This decrease can be determined by different models developed for bottleneck analyses. Three statistical analyses known as the sign, standardized differences, and Wilcoxon sign rank test may be utilized under the infinite allele model (IAM), two-phased mutation model (TPM), and stepwise mutation model (SMM) to decide whether populations have faced a recent bottleneck (Cornuet and Luikart 1996). SMM approach utilises the changes in fragment length including increases and decreases; TPM benefits from mutations which are not of change in allele size, whereas heterozygosity is considered as a function of consistent changes in allele frequency in a given population (Ullah et al. 2021). On the other hand, the TreeMix algorithm is available to detect population splits and migration events among animal populations sharing a common breeding history (Pickrell and Pritchard 2012).

The genetic bottleneck in native Turkish sheep breeds has been exclusively assessed via microsatellite markers (Akay et al. 2020; Ata et al. 2020; Ozmen et al. 2020; Kabasakal 2023), while high-density SNP data were utilized to reveal migration events in native Turkish sheep and cattle populations (Demir et al. 2023; Karsli 2024). However, a recent study confirmed that microsatellite markers were also efficient molecular genotyping methods to investigate migration events in livestock species (Demir 2024). In this regard, a previous study carried out by Karsli et al. (2020) has identified the genetic diversity and population structure of GKR, KRK, KNG, and NRD sheep breeds, while genetic bottleneck and migration events were not evaluated. By extending this genetic data, the current study aims to evaluate bottleneck and migration events in four native sheep breeds raised in Türkiye based on 28 microsatellite loci and to discuss the results obtained in terms of the sustainable use of these breeds.

## Material and methods

### Animal sampling and molecular genotyping

To obtain deeper knowledge on the Akkraman breed, its four ecotypes (GKR, KRK, KNG, and NRD) were studied for genetic bottleneck and migration events analyses. Baes on oral interviews with breeders, a total of 120 unrelated blood samples from four different native sheep breeds (30 samples from each breed) were used in the completed study. Blood samples of KNG and GKR breeds were collected from Sivas and Antalya provinces, respectively, while blood samples of NRD and KRK breeds were obtained from Van province. Genomic DNA isolation from blood samples was performed according to the salting-out protocol described by Miller et al. (1988). Agarose gel electrophoresis (1%) was performed to confirm whether the DNA isolation process was successful. The quantity and purity of DNA were determined using a spectrophotometer (NanoDrop-SD 1000). Following DNA extraction, the DNA concentration was adjusted to 50 ng/µL for the PCR process.

The animals sampled in this study were previously genotyped via FAO recommended 21 microsatellite markers such as (*OarJMP58, SPS115, MCM527, HSC, BM8125, ILTS28, OarJMP29, ILTS11, ETH10, OarFCB304, CSRD247, MAF214, MAF65, BM1824, SRCRSP9, CSSM66, SRCRSP1, BM1818, MAF70, SRCRSP5,* and *D5S1*) by Karsli et al. (2020) who did not analyze genetic bottleneck and migration events. In this study, however, this genetic data was extended by genotyping these animals with an additional seven microsatellite markers (*HUJ616, OarFCB128, OarVH72, DYMS1, MCM140, OarFCB193,* and *MAF33*). As a result, a total of 28 microsatellite loci were utilized to reveal genetic bottleneck and migration events in four native sheep breeds reared in Türkiye.

Polymerase chain reactions (PCR) were carried out in a total volume of 25 μL of PCR mixture. PCR mixture consisted of 2.5 μL genomic DNA (50 ng/μL), 1.2 μL HQ buffer (Geneall), 2 μL dNTPs (2.5 mM/μL), 0.25 μL of each primer (10 pmol/μL), 0.4 μL (2.5 U/μL) Taq polymerase (Geneall), and was increased to 25 μL with deionized water. The PCR was performed with an initial denaturation at 95 °C for 5 min, followed by 30 cycles of denaturation at 94 °C for 45 s, annealing at 50 °C–60 °C (based on each primer pair) for 45 s, and extension at 72 °C for 45 s. The final extension was optimized at 72 °C for 5 min.

The sizes of PCR products were determined using a 96-automated capillary electrophoresis system (Advanced Analytical Technologies-AATI, Ames, Iowa, USA). The capillary conditioning solution, inlet buffer, separation gel, and 35–500 bp marker were all used according to the manufacturer's instructions. After capillary electrophoresis, allele sizes were scored by using PROSize® 2.0 version 1.3.1.1 (AATI, Ames, Iowa, USA).

### Statistical analysis

Bottleneck v1.1.2.02 (Piry et al. 1999) was run for sign, standardized differences, and the Wilcoxon sign rank test under the IAM, SMM, and TPM model as well as the mode-shift indicator to analyze the bottleneck effect in four native Turkish sheep breeds. In order to visualize L-shaped distributions of allele frequency, the results of the mode-shift indicator were processed with the *plot* function implemented in R software (https://www.r-project.org). LDNe software (Waples and Do 2008) was run with default parameters to estimate the effective population size (Ne) based on three levels of the lowest allele frequency parameter (0.01, 0.02, and 0.05) across studied sheep populations. The results of the Ne analysis were processed by the ggplot2 package (Wickham 2011) implemented in R software (https://www.r-project.org) for visualization.

TreeMix software (Pickrell and Pritchard 2012) was run with pre-defined parameters such as up to 5 migration events with 20 iterations and 1000 bootstrap values to reveal population splits and gene flow among four native Turkish sheep breeds. The optimal number of migration events was determined by using the OptM package (Fitak 2021) implemented in the R software (https://www.r-project.org) via the Evanno approach. Moreover, the migration rate per event was calculated by the Bayesass v1.3 (Wilson and Rannala 2003) with a pre-defined value of iterations (1000). The results of migration events were visualized by using the *BITE* package (Milanesi et al. 2017) implemented in R software (https://www.r-project.org).

## Results

### Genetic bottleneck effects

In this study, mutation drift equilibrium-based three statistical approaches were utilized to determine whether the effective population sizes have been sustained or reduced in the recent past among four native Turkish sheep populations. The significance of these approaches has changed, with the expected number of loci with heterozygosity excess ranging from 12.32 (under the SMM model in KRK) to 12.55 (under the IAM model in KRK and KNG) (Table 1).

**Table 1:** Calculated values for mutation drift equilibrium in four native Turkish sheep populations under various models and approaches

Breed	Mutation model	Sign test			Standardized differences test		WS rank test (one tail for He)
		Hee	He	P	T2	P	P
GKR	IAM	12.49	21	0.00002*	5.131	0.00000*	1.00000
	SMM	12.37	15	0.17286	1.278	0.10069	0.94839
	TPM	12.48	15	0.00028*	3.787	0.00000*	1.00000
NRD	IAM	12.53	20	0.00029*	4.753	0.00000*	1.00000
	SMM	12.48	14	0.32982	0.499	0.30872	0.85613
	TPM	12.47	18	0.00948*	3.317	0.00046*	0.99974
KRK	IAM	12.55	19	0.00222*	3.392	0.00035*	0.99970
	SMM	12.32	11	0.35457	−2.803	0.00253*	0.10784
	TPM	12.49	17	0.03334	1.221	0.11097	0.99365
KNG	IAM	12.55	21	0.00002*	5.221	0.00000*	1.00000
	SMM	12.42	19	0.00188*	1.773	0.03809*	0.99686
	TPM	12.49	19	0.00207*	4.073	0.00002*	1.00000

GKR: Güney Karaman; NRD: Norduz; KRK: Karakaş; KNG: Kangal; Hee: expected number of loci with heterozygosity excess; He: number of loci with heterozygosity excess; T2: test 2; WS: Wilcoxon-sign; IAM: infinite allele model; SMM: stepwise mutation model; TPM: two-phase model; P: probability value for heterozygosity excess; *: Rejection of null hypothesis (*P* < 0.05)

The sign test revealed a higher expected number of loci with heterozygosity excess than the actual number of loci with heterozygosity excess in all mutation models across all populations except for the SMM model in the KRK population (Table 1). Similarly, a negative T2 value (−2.803) for the standardized differences test was detected only in the SMM model in the KRK population.

**Fig. 1:**
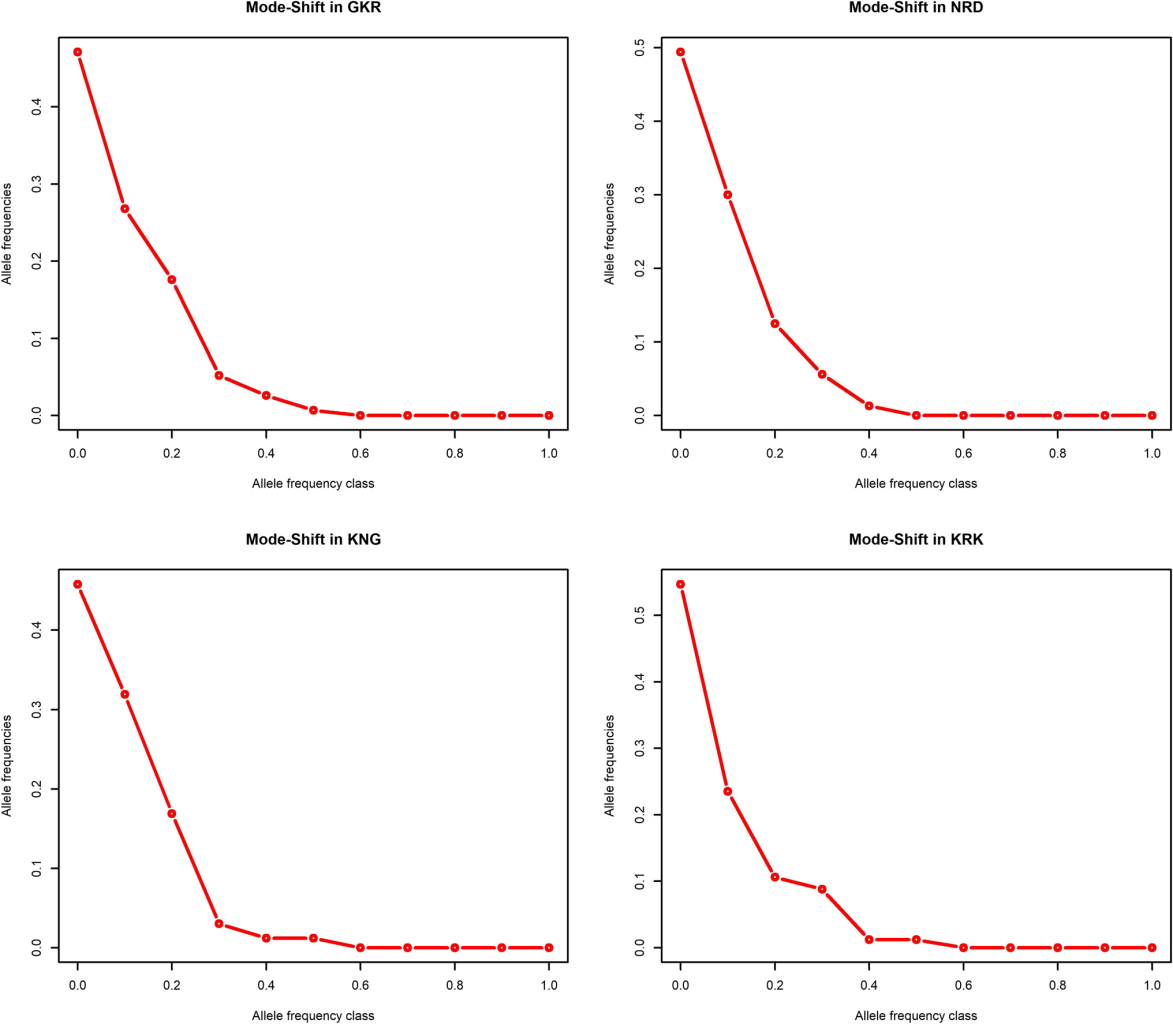
Mode-shift test for qualitative assessment of genetic bottleneck in native sheep populations

In this study, the probability values for the Wilcoxon sign rank test under the TPM model were estimated at 1.00 for GK and KNG breeds, while it was 0.99 for NRD and KRK breeds. Under the SMM model, this value was calculated as 0.94, 0.85, 0.11, and 0.99 for GKR, NRD, KRK, and KNG breeds, respectively (Table 1). The null hypothesis was not rejected in four native sheep populations based on some mutation models with sign and standardized differences tests (Table 1). On the other hand, the Wilcoxon rank test detected no genetic bottleneck effects in four native Turkish sheep populations under all three mutation models. Similarly, being a qualitative method, the mode-shift indicator showed the normal L-shaped distribution of allele frequencies which also suggested the lack of bottleneck effects in four native Turkish sheep breeds (Fig. 1).

### Linkage disequilibrium-based effective population size

**Fig. 2:**
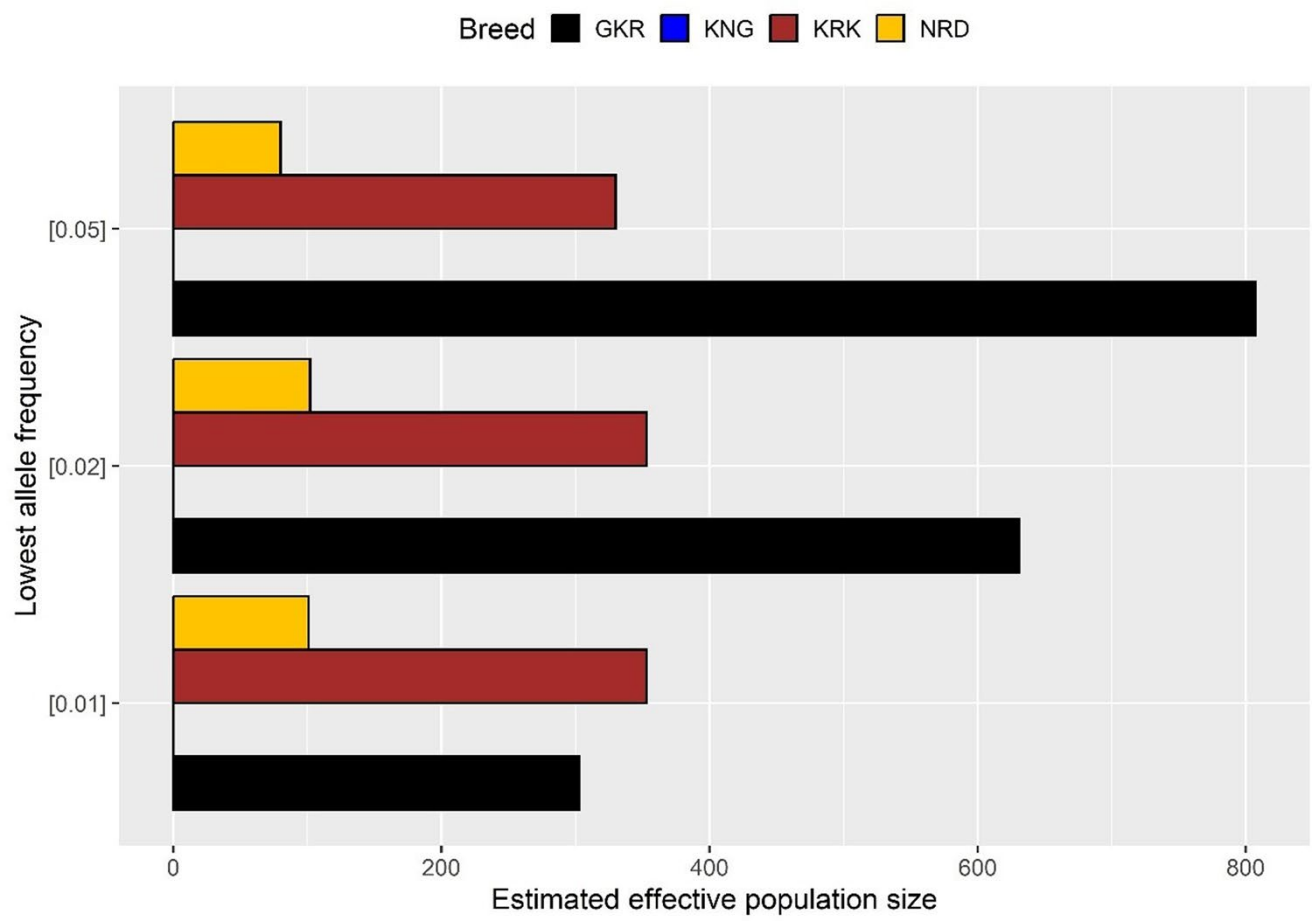
Number of effective population size based on different lowest allele frequency thresholds

The results of Ne analysis showed consistency with genetic bottleneck analysis in which the number of effective population size was recorded as “infinite” for the KNG breed. On the other hand, the lowest and highest Ne values were observed in NRD (80 individuals), and GKR (820 individuals) when the lowest allele frequency was 0.05. A similar trend in effective population sizes was observed when the lowest allele frequency decreased to 0.02, and 0.01 (Fig. 2).

### Migration analysis

**Fig. 3:**
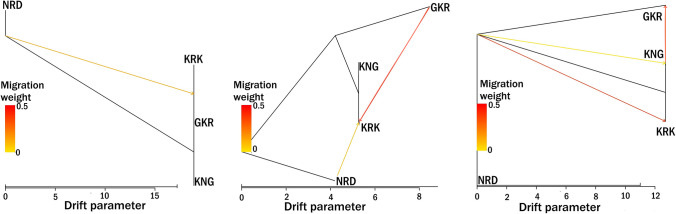
Maximum-likelihood tree based on TreeMix algorithm with one to three migration events across the studied sheep populations

The TreeMix algorithm was utilized to assess migration events among native Turkish sheep populations (Fig. 3) in which the delta m value based on the Evanno approach revealed that the optimal number of migration events among four native Turkish sheep breeds was 3.

In the case of one event, a migration from NRD to the KRK-GKR clade was observed (Fig. 3). The migration rate from NRD to KRK-GKR clade was calculated as 0.0096 (data not shown). When two events were taken into consideration, there was a migration from both NRD and GKR to the KRK breed with a migration rate of 0.0099. In the best delta m value (3), another migration from KNG to GKR was drawn with a 0.0176 rate (Fig. 3).

## Discussion

This study revealed inconsistencies among statistical approaches and mutation models used for bottleneck detection. Because genetic bottleneck effects were detected via some mutation models with the sign and standardized differences tests, whereas the Wilcoxon sign test and mode-shift indicator confirmed that four native Turkish sheep breeds have not experienced a genetic bottleneck in the recent past. Inconsistent results seem to occur due to different assumptions behind the statistical approaches. Similar inconsistent results were also detected in the literature. For example, a recent genetic bottleneck was reported in the Poonchi sheep breed genotyped with six microsatellite markers in terms of sign and standardized differences test under the IAM and TPM model, while the population was non-bottlenecked regarding the SMM mutation model (Azhar et al. 2020). Similarly, a study utilizing a total of 31 microsatellite loci on three sheep breeds reared in Pakistan showed a genetic bottleneck in the Michni breed via SMM model, while the population was under mutation drift equilibrium by IAM and TPM models (Ibrahim et al. 2018). Another study using 25 microsatellite markers revealed that Tibetian sheep in India have not experienced a recent genetic bottleneck according to the sign test with all mutation models, whereas a significant deviation from mutation drift equilibrium was reported via the Wilcoxon sign rank test under IAM and SMM mutation models (Sharma et al. 2016). Moreover, in another study in which a total of 103 Karacabey Merino sheep were genotyped with 14 microsatellite markers, the probability values for heterozygosity excess were lower than 0.05 in terms of the sign and standardized differences tests under TPM and SMM model as well as the Wilcoxon sign test under IAM mutation model, while normal L-shaped distribution was declared by mode-shift indicator (Kabasakal 2023). These inconsistencies between statistical approaches in terms of genetic bottleneck analysis have been evaluated by several studies which have emphasized that the Wilcoxon test under the TPM mutation model, as well as the mode-shift indicator, are the most accurate approaches to investigate genetic bottleneck in livestock species (Vohra et al. 2017; Azhar et al. 2020). Regarding this criterion, it could be concluded that four native Turkish sheep breeds have not experienced a recent genetic bottleneck and maintained their effective population size. Indeed, the number of estimated effective population size for GKR, KRK, and NRD sheep populations were higher than the actual number of sampled animals, while negative and “infinite” values of Ne were estimated for the KNG breed. As mentioned by previous studies (Do et al. 2014; Demir 2024), negative values of Ne, together with the “infinite” upper confidence interval values, could be observed when the actual amount of the sampling error can be greater than the estimated value. Thus, future studies should adopt improved sampling strategies to more accurately estimate the effective population size of the KNG breed. Several studies also found similar findings regarding the genetic bottleneck analysis. For example, a normal L-shaped curve distribution of allele frequencies was reported in 180 head sheep raised in different regions of Türkiye and Algeria by fifteen microsatellite markers (Ata et al. 2020). Another study focusing on a total of 119 animals genotyped in terms of sixteen microsatellite markers indicated that GKR has not experienced serious demographic bottlenecks according to the mode-shift indicator and the Wilcoxon test under the TPM mutation model (Akay et al. 2020). Besides, another study conducted by Ozmen et al. (2020) revealed that KNG, KRK, and NRD have maintained their effective population size according to three mutation models.

Both current and previous studies confirm that native Turkish sheep breeds have lacked genetic bottleneck indicating that they still have maintained their effective population size. However, it is known that the number of native genetic resources including cattle, goats, and sheep is at the declining trend in Türkiye. Therefore, genetic bottleneck analysis should be repeated periodically in order for taking precautions for sustainable production in the future.

Recent microsatellite-based studies have confirmed that especially NRD breed has become genetically different from AKR, GKR, KRK, and KNG populations (Yilmaz et al. 2014; Karsli et al. 2020) via population structure analysis. However, no microsatellite-based studies were available in the literature to investigate population splits and gene flow by the TreeMix algorithm in native Turkish sheep breeds. In this study, however, TreeMix-based migration events analysis revealed fundamental hints about the genetic background of four sheep populations. As known, NRD has been raised in a limited region of Türkiye (Van province) for a long time. This geographic isolation may hinder gene flow from other breeds into NRD. Indeed, a genetic migration from NRD to GKR, KNG, and KRK was observed, whereas no migration from other populations into NRD was detected. This is the first study to confirm the genetic distinctiveness of NRD from other native Turkish sheep breeds using the TreeMix algorithm. However, this kind of breeding will probably result in decreased genetic diversity and bottleneck effects in the future. Therefore, comprehensive conservation programs should be initiated to eliminate the risk of decreases in genetic diversity in NRD.

In conclusion, this study revealed no genetic bottleneck in four Anatolian sheep breeds based on the mode-shift indicator and IAM, SMM, and TPM mutation models under the Wilcoxon sign rank test. Similarly, the estimated population sizes were higher than the number of sampled animals across four sheep populations. Still, these breeds should be periodically monitored via microsatellite and high-density SNP data regarding genetic bottlenecks to take precautions for sustainable production in the future.

TreeMix-based migration events analysis clarifies that no migration from other populations into NRD is available, which implies that NRD is genetically distinct from other native Turkish sheep breeds due to geographic isolation and pure breeding practices by the farmers. To preserve genetic diversity, particularly in NRD, periodic monitoring and well-designed conservation strategies are essential.

## Data Availability

The datasets used in this study for seven microsatellite markers (*HUJ616, OarFCB128, OarVH72, DYMS1, MCM140, OarFCB193,* and *MAF33*) are available upon request via a Material Transfer Agreement signed by the corresponding author for scientific purposes only.
